# Coffin-Siris Syndrome and SMARCB1 Mutation Presenting With Schwannomatosis: A Case Report and Literature Review

**DOI:** 10.7759/cureus.67333

**Published:** 2024-08-20

**Authors:** Julia E Gallagher, Daryoush Saeed-Vafa, Marilyn M Bui, Rikesh Makanji

**Affiliations:** 1 Department of Medicine, University of South Florida Health Morsani College of Medicine, Tampa, USA; 2 Department of Pathology, Moffitt Cancer Center and Research Institute, Tampa, USA; 3 Department of Diagnostic Imaging and Interventional Radiology, Moffitt Cancer Center and Research Institute, Tampa, USA

**Keywords:** smarcb1, soft tissue tumor, diagnosis, biomarker, schwannomatosis, coffin-siris syndrome

## Abstract

Coffin-Siris syndrome (CSS) is a rare genetic condition associated with mutations in genes responsible for the modulation of gene expression and chromatin remodeling. Patients with CSS commonly present with congenital anomalies, intellectual disabilities, and developmental delays. We describe a case of a 28-year-old woman with a confirmed diagnosis of CSS and *SMARCB1 *mutation who presents with multiple schwannomas and an intra-abdominal neurofibroma. The patient underwent embolization and resection of an enlarging, symptomatic schwannoma of her left medial upper arm. In detailing the patient’s presentation, this case report underscores the association between *SMARCB1 *mutations, CSS, and tumorigenesis.

## Introduction

Coffin-Siris syndrome (CSS) is a rare genetic syndrome that commonly presents with developmental or cognitive delays, coarse facial features, aplasia or hypoplasia of the distal phalanx or nail of the fifth digit, congenital abnormalities, hypertrichosis, feeding challenges, and slow growth [1]. Although the prevalence of CSS is unknown, fewer than 300 cases have been reported [2]. CSS arises from mutations in genes that encode the subunits of the Brahma-related gene 1 (BRG1)- and Brahma (BRM)-associated factor (BAF) complex, which are responsible for chromatin remodeling and modulation of gene expression [1,3]. Mutations in *ARID1A*, *ARID1B*, *ARID2*, *DPF2*, *SMARCA4*, *SMARCB1*, *SMARCC2*, *SMARCE1*, *SOX11*, and *SOX4* are linked to CSS [2]. Pathogenic variation in *SMARCB1 *constitutes an estimated 7-12% of CSS cases and predominantly results from *de novo* mutations [1,3].

In addition to CSS, *SMARCB1*, which is located at 22q11.23 centromeric to the *NF2 *allele, has been associated with tumorigenesis [4]. Notably, individuals with the *SMARCB1 *pathogenic variant have an increased risk of developing schwannomatosis [3]. Patients with schwannomatosis develop multiple peripheral schwannomas and, less frequently, meningiomas from inactivating mutations of *SMARCB1 *or *LZTR1 *[5]. Although mutations in *SMARCB1 *are observed in both CSS and schwannomatosis, the literature notes one previous case of an individual with a *SMARCB1 *mutation diagnosed with CSS and schwannomatosis [6]. This case report discusses the clinical presentation, imaging, and pathology of a patient who presents with schwannomatosis and a diagnosis of CSS with a *SMARCB1 *mutation.

## Case presentation

A 28-year-old woman with a history of CSS, grand-mal seizures, Wolff-Parkinson-White syndrome, and chronic kidney disease presented with multiple schwannomas and an intra-abdominal neurofibroma. The patient received a suspected CSS diagnosis in infancy following evaluation by medical genetics. Clinical findings revealed stunted growth and weight, coarse facial features, small fifth fingernails, fifth toenails and phalanges, sparse scalp hair, and hypertrichosis of the forehead and spine. She has required a gastrojejunostomy (GJ) tube since infancy due to feeding difficulties. Continued evaluation by medical genetics noted developmental delays and intellectual disability during the patient’s childhood and adolescence. The patient is nonverbal and dependent on a wheelchair despite learning to walk at the age of six. Additionally, she has hypoplasia of the corpus callosum and a Dandy-Walker cyst. Genetic testing confirmed the patient’s CSS diagnosis, revealing a Lys364 deletion of the *SMARCB1 *protein. The patient’s biological parents and full siblings are not suspected to have CSS; thus, the patient is believed to have a *de novo* mutation.

**Figure 1:**
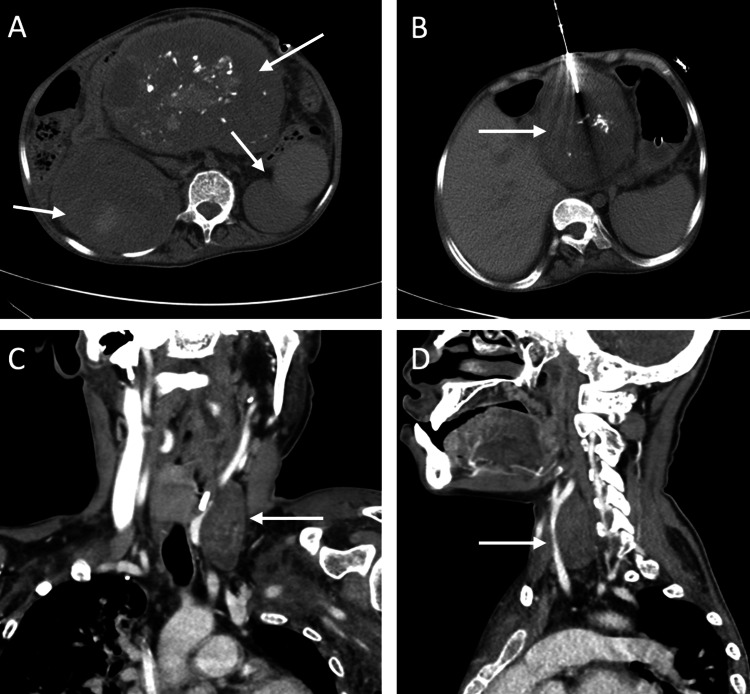
(A) A contrast-enhanced CT scan demonstrates heterogeneous mesenteric and perirenal masses, with the mesenteric mass showing calcifications within. (B) CT-guided biopsy confirming calcifications within the mesenteric mass. (C) Coronal neck CT with intravenous contrast demonstrates a left carotid mass abutting and displacing the left common carotid artery and causing a mass effect upon the left internal jugular vein. (D) Sagittal neck CT with intravenous contrast demonstrates a left carotid mass abutting and displacing the left common carotid artery and causing a mass effect upon the left internal jugular vein.

In 2008, the patient was found to have a retropancreatic mass in the porta hepatis while undergoing preoperative imaging for scoliosis surgery. A biopsy of this mass revealed a single plexiform neurofibroma. Surgical resection of the neurofibroma was attempted yet was unsuccessful due to hemorrhaging. Ten years later, the 25-year-old patient was hospitalized following a fall; during her hospitalization, full-body magnetic resonance imaging (MRI) and computed tomography (CT) scans revealed multiple masses: a mass on the superior pole of her right kidney, a lesion near her left common carotid artery, a large mass in her left upper arm, and the previously discovered upper-mid abdominal mass (Figure 1).

The patient’s mother reports that the left upper arm mass has been present for several years but has recently become painful and increased in size. A physical exam of this lesion revealed a large soft tissue mass located in the medial upper arm with no overlying skin changes.

**Figure 2:**
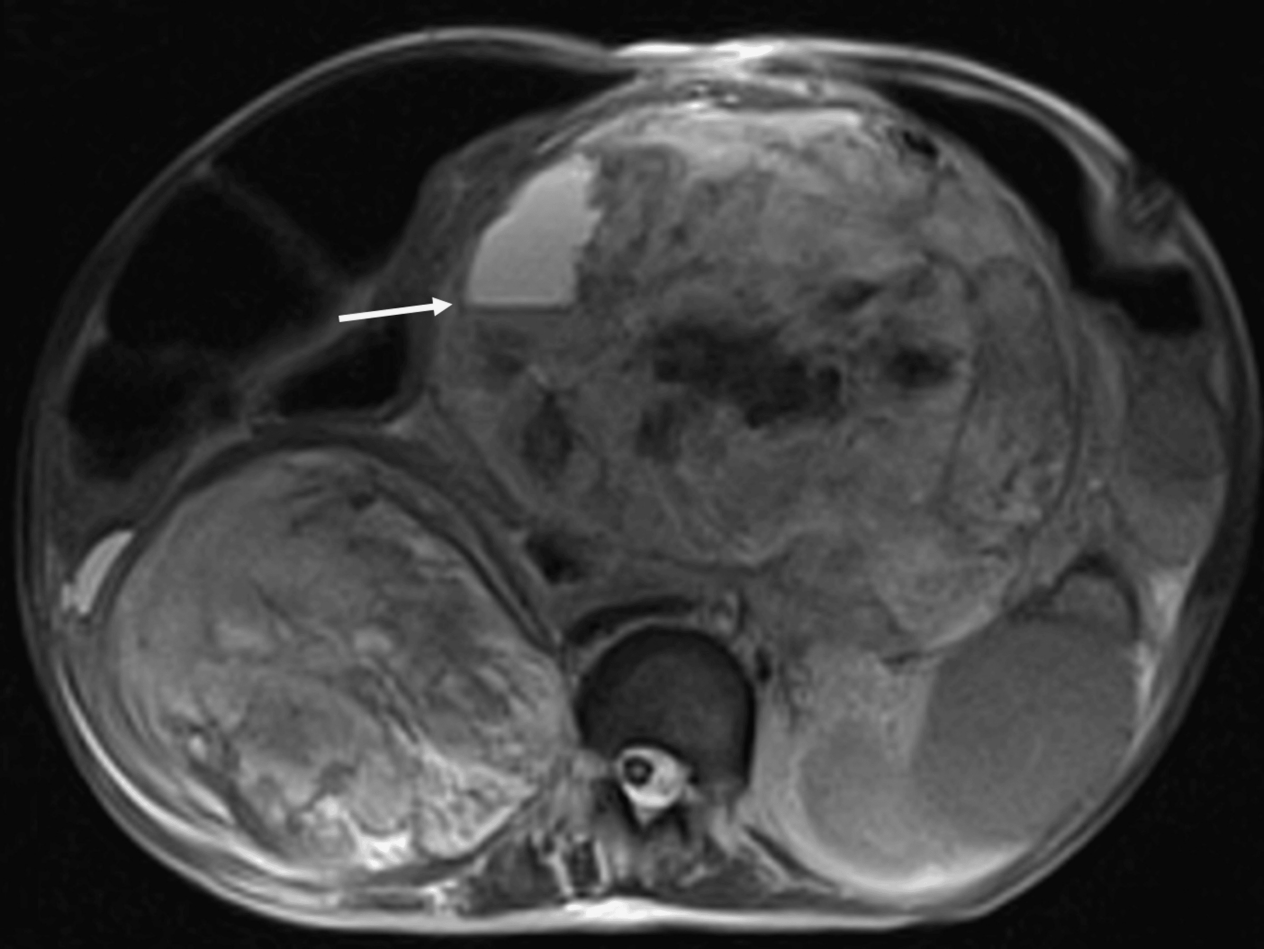
T2-weighted fat-saturated axial image of the abdomen demonstrating fluid levels within the mesenteric mass.

At this time, an MRI of the right renal and upper-mid abdominal masses noted concern for malignancy. The 11 cm right renal mass demonstrated invasion of the posterior abdominal wall in the right T12-L1 neural foramen. Additionally, the 18 cm upper-mid abdomen mass was mostly fibrous but displayed some areas that were suspicious for malignant degeneration (Figure 2).

Histopathology of these masses, however, confirmed benign peripheral sheath tumors.

In 2019, the patient underwent a left upper extremity venous ultrasound (US) to evaluate the left upper arm and lower neck masses. Imaging revealed a 2.2 × 2.2 × 3.2 cm mass posterior to the left internal jugular vein and posterior-lateral to the common carotid artery. The mass was hypervascular and extended to the thoracic inlet. The US further identified a 9.6 × 9.3 × 9.7 cm soft tissue mass adjacent to the metadiaphyseal region of the left humerus that displayed internal vascularity and cystic changes.

**Figure 3:**
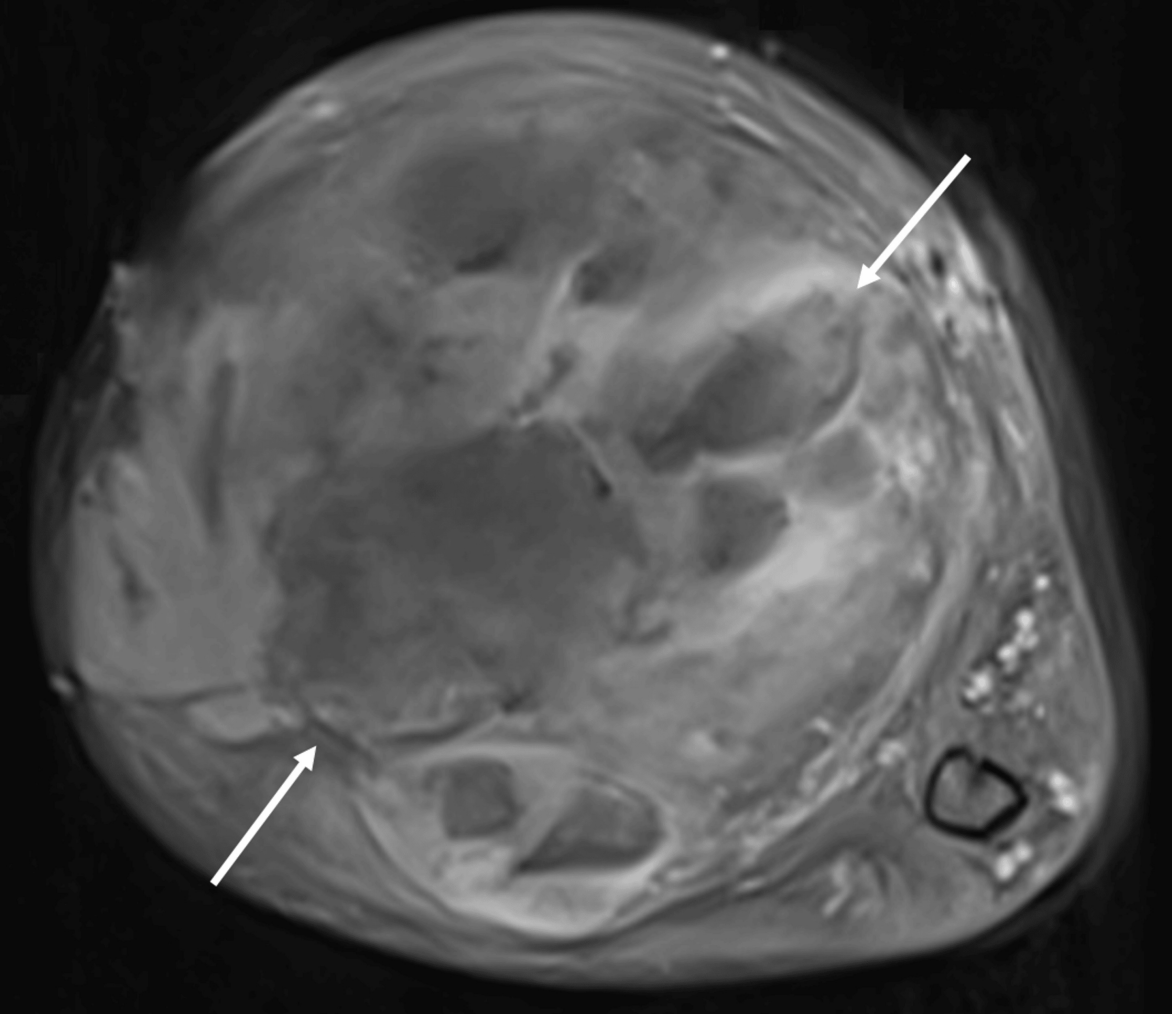
T1 axial post-contrast fat-saturated MRI of the upper arm demonstrates a heterogeneous soft tissue mass with sites of enhancement noted.

A series of imaging studies were performed between 2019 and 2021 to evaluate the increasing size of the upper arm mass. One month following the patient’s US, multiplanar multisequence magnetic resonance (MR) images were obtained through MRI before and after administration of 3 cc intravenous Gadavist contrast. T1-weighted imaging noted a heterogeneous soft tissue mass measuring 10.5 × 8 × 12.4 cm (Figure 3).

Imaging did not note suspicion of osseous involvement or marrow lesions. A CT angiography of the left upper extremity was performed three months later, which demonstrated a 13 × 10 × 10 cm tumor. In 2021, MRI with and without IV contrast noted an increase in the mass’s size to 15.2 × 11.9 cm from the CT’s measurement of 10 × 10 cm. There was concern for malignancy due to the tumor’s growing size. Histological analysis, however, confirmed a peripheral nerve sheath tumor consisting of a proliferation of bland spindled cells with wavy serpentine nuclei.

**Figure 4:**
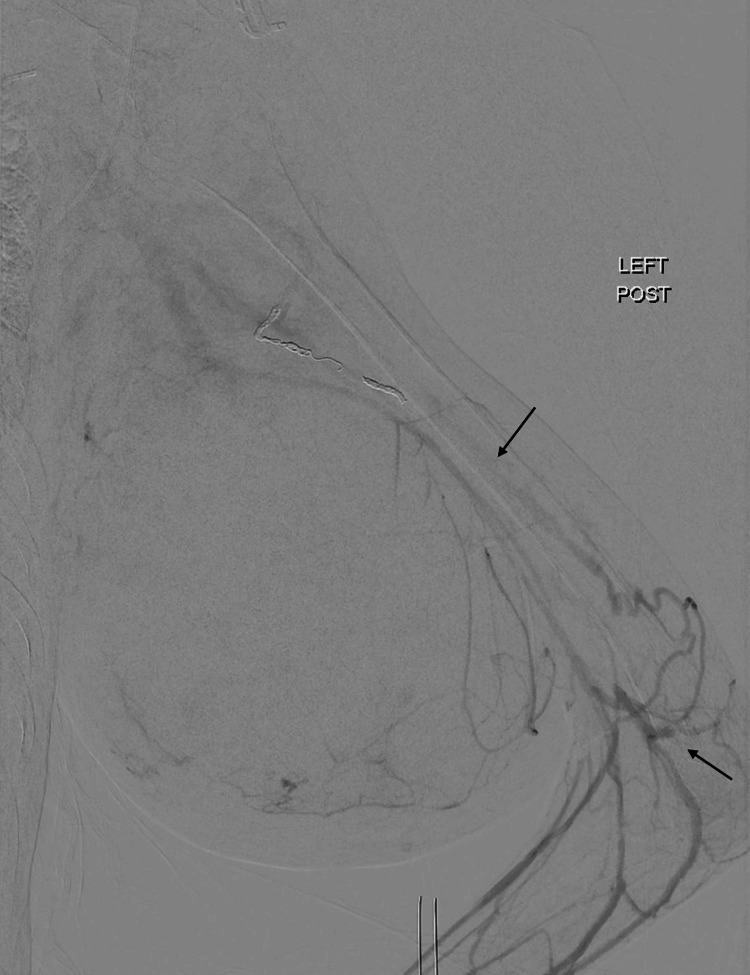
Angiographic images demonstrate a left upper extremity hypervascular tumor status after successful multivessel embolization using particles and coils. Postembolization angiogram showed approximately 80% decrease in arterial blood supply to the tumor.

The patient’s case was discussed twice by the tumor board, who decided against systemic treatment because the patient did not have a targetable mutation. It was decided that the patient would receive surgical management of the left upper arm mass. In 2022, resection of her left medial upper arm soft tissue mass was performed with preoperative embolization of the left upper extremity (Figure 4).

**Figure 5:**
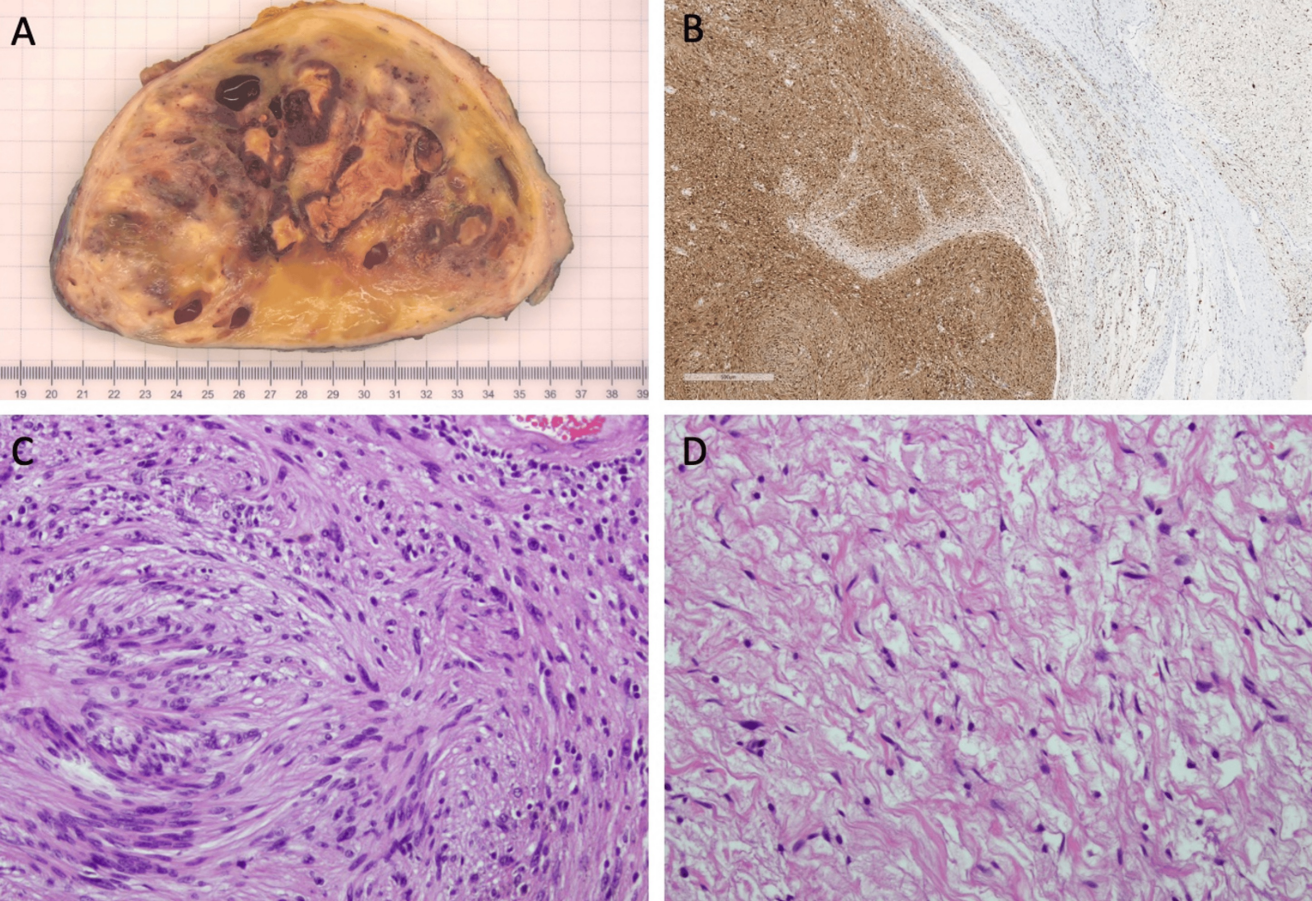
(A) Gross specimen: benign peripheral nerve sheath tumor that is predominantly schwannoma with focal features of neurofibroma. Areas of degenerative changes are seen in the center. (B) S-100 slide images at ×200 magnification highlight the schwannoma on the left and the neurofibroma on the right. (C) Hematoxylin and eosin-stained slide images at ×200 magnification compatible with schwannoma. (D) Hematoxylin and eosin-stained slide images at ×200 magnification compatible with neurofibroma.

The final resected mass measured 20 × 14 × 10 cm. Histologic evaluation diagnosed the left medial upper arm soft tissue mass as a benign peripheral nerve sheath tumor that is predominantly schwannoma with focal features of neurofibroma (Figure 5).

Additionally, targeted next-generation sequencing (NGS) was completed on the mass, confirming a *SMARCB1 *p.K364del mutation. NGS was performed with the Illumina TruSight Tumor 170 (Illumina Inc., San Diego, CA) platform, a hybrid-capture 170-gene panel designed to identify clinically important small variants and copy number variants by DNA-based testing while simultaneously identifying splice variants and fusion by RNA-based testing of tissue from solid tumors [7].

## Discussion

Mutations of the *SMARCB1 *protein subunit of the BAF complex are implicated in both CSS and schwannomatosis [1,8]. However, the exact etiologies of these syndromes are not completely understood. The *SMARCB1 *pathogenic variant of CSS has an autosomal dominant inheritance pattern that leads to non-truncating mutations that are missense or in-frame deletions [1]. It is suggested that the *SMARCB1 *pathogenic variant in CSS patients arises from a *de novo *germline mutation that results in a dominant-negative or gain-of-function effect [1]. Contrastingly, *SMARCB1 *pathogenic variations in schwannomatosis patients are categorized as familial or sporadic, comprising approximately 48% and 10% of schwannomatosis cases, respectively [6]. Germline *SMARCB1 *mutations are observed in approximately 50% of familial cases and 10% of sporadic cases [5]. Current studies hypothesize that schwannomatosis arises from a four-hit, three-step model [4,9]. The first hit results in a *SMARCB1 *germline mutation (step 1). The subsequent somatic hits occur in the schwannoma: two hits lead to the loss of the region of chromosome 22 containing the *SMARCB1 *and *NF2 *alleles (step 2), and the final hit mutates the remaining *NF2 *allele (step 3). Despite this model, the genetic cause of schwannomatosis is uncertain in approximately 60% of sporadic cases and 14% of familial cases [5,10].

Although *SMARCB1 *is mutated in schwannomatosis and CSS, the literature describes one confirmed case of a CSS patient presenting with schwannomatosis and a *SMARCB1 *pathogenic variant. The case report describes a 33-year-old man diagnosed with CSS who presented with schwannomas in his tongue, neck, spine, and extremities at the age of 26 [6]. The patient underwent genetic analysis, which revealed a germline *SMARCB1 *missense mutation in exon 9, c.1121G>A (p.Arg374Gln) with somatic mutations of *SMARCB1 *and *NF2 *in his biopsied tumors. The literature additionally describes a case of a 28-year-old female with a missense c.1120C>T (p.R374W) *SMARCB1 *mutation in exon 9, who had a history of multiple schwannomas and mild tricuspid, mitral, and aortic valvular insufficiency. This case report speculates that the patient’s cardiac abnormalities signified a partially expressed case of CSS; however, her clinical presentation did not meet the diagnostic criteria for a CSS diagnosis [11]. Our patient supports the theory that the initial hit for schwannomatosis results from a germline *SMARCB1 *mutation. However, the *SMARCB1 *pathogenic variant in our patient stems from a germline in-frame deletion (*SMARCB1 *(INI) mutation c1091del; LYS364 DEL). Thus, both patients developed schwannomas despite having different *SMARCB1 *pathogenic variants. A previous study conducted by Kosho et al., 2014 identified the Lys364 deletion as the only recurrent *SMARCB1 *mutation in a group of 13 CSS patients with the *SMARCB1 *pathogenic variant. However, none of these individuals exhibited schwannomatosis [12].

## Conclusions

This patient with a germline *SMARCB1* mutation and confirmed CSS diagnosis developed schwannomatosis. She has no known family history of CSS and is thus suspected to have a *de novo* *SMARCB1 *mutation. This patient initially presented with several peripheral nerve sheath tumors, including an enlarging schwannoma of her left medial upper arm. Due to the lack of a targetable mutation, the patient did not receive systemic therapy but underwent pre-operative embolization and surgical resection of her enlarging left upper arm mass. Further investigation is needed to elucidate the genetic mechanism behind the BAF complex’s role in tumorigenesis and genetic syndromes. It is unclear how mutations of *SMARCB1 *lead to tumorigenesis or differences in phenotypes amongst CSS patients. Ultimately, additional research on the BAF complex is needed to better understand the development of these syndromes.
